# New York Letter

**Published:** 1882-01

**Authors:** 


### Domestic Correspondence.

### ARTICLE VIII.

### NEW YORK LETTER.

EDITORS OF THE CHICAGO MEDICAL JOURNAL AND EXAMINER.—New York is not falling away at all in the activity among its medical societies. A new one has recently been organized under the title of the Materia Medica Society. It will devote itself to the subject of drugs and therapeutics. The gentlemen who have been most energetic in getting it up are wide-awake and competent persons. Some of them, such as Dr. Piffard and Dr. Castle, have paid more than ordinary attention to therapeutics. The former has some peculiar views, which, if not homœopathic, are rather heretical. We have a Therapeutical Society in the city, and it would seem rather unnecessary to have another whose objects must be very nearly alike. It is said, however, that the Therapeutical Society, though it has done some good work, is now moribund, or at least very inactive.

At a meeting of the Medico-Legal Society some time ago, Dr. T. D. Crothers read a paper on Trance-States in Inebriates. The speaker contributed a large amount of valuable clinical matter to the subject of inebriety. This he considers as unquestionably a disease. During it, there not very rarely occur conditions of blanks in memory and consciousness. He terms these trance-states. The patients act then like automata and do things for which they are not at all responsible. Hence the medico-legal interest. The state described is a very curious and interesting one. The society met again last week. Dr. Johnson, of Brooklyn, read a paper on the Medico-legal Relations of Anæsthetics. One of the most interesting points brought out was whether it is justifiable to give an alleged criminal an anæsthetic in order to make him confess. Of course, the opinion was given that this should not be done, on the legal ground that we have no right to make a man criminate himself. As physicians, the question of most interest is whether such a process as chloroforming could bring out any confession that would be of real value. There is yet no evidence that any testimony, in itself trustworthy, could be elicited in such a way. The discussion on the paper was postponed, however, in order that the Society might elect officers and have a supper. There was a dead-lock when it came to the election of President,—but nothing interfered with the supper. Indeed the novelty of mental harmony and abundant diet seemed so pleasing that one enthusiastic member moved that the annual supper be held every month. This was acceded to in so far as to vote for another supper at the next monthly meeting. The Medico-Legal Society is a kind of hodge-podge of good and bad material; of doctors who want to be experts, and lawyers who want to talk. It has in it, however, some most excellent men in both professions. It has also a large membership, an excellent library, and it publishes a regular bulletin of its proceedings. There has been a lack of harmony and rather a falling back in its work of late. Dr. Hammond withdrew last spring because he thought himself unfairly treated when he presented the subject of mesmerism at that time—and there were some other disaffections. The coming election of officers is expected to restore peace and prosperity.

At a meeting of the New York Academy of Medicine, recently, Dr. Taylor read a long paper on the subject of reflex disturbances of the sexual organs in women. The gist of the paper was to the effect that unmarried women are sometimes subject to very severe constitutional disturbances, due to the fact that the sexual function is not employed. Furthermore, such women may suffer and be chronic invalids from this cause without at all knowing what is the matter. The clinical facts cited in proof of this statement were somewhat startling, when taken alone, as they went to show that the sexual feeling in woman is very powerful, and that the inability to gratify it is sometimes attended with very serious consequences. Dr. Taylor said, however, that there was a remedy for the threatened danger. This remedy lay in active and regular physical exercise, and in a systematic employment of the mind. The discussion at the close of the paper was much more personal than scientific, and in no way added to the interest of the meeting. Dr. Fordyce Barker referred to the fact that the subject was a delicate one, but he thought it ought to be carefully discussed. Dr. Lewis Sayre then rose, and in his most vigorous style denounced the character of the paper, and said that he did not think the facts given ought to go out endorsed by the Academy. The reader of the paper had virtually intimated that single women are in danger of losing their health if the sexual function were not employed. If medical men endorsed such a view, and it should get abroad, what an incentive to, or excuse for, immorality would be given. It was said, in reply to this, that Dr. Taylor had only stated that in rare cases women might suffer from the cause mentioned. If this were so, the fact should be known to medical men and taken into account by them. The preventive measures of regular bodily and mental exercise could be employed.

You are aware, probably, that Dr. Wm. J. Morton, of this city, has purchased the *Journal of Mental and Nervous Diseases*, heretofore edited so ably by Dr. Jewell, of your city. Dr. Morton will now assume the sole editorship of it.

The proposed new weekly medical journal to be started in opposition to the *Record* is not to be published,—at least this is the latest report. Not enough money could be raised for it. In part, perhaps, to supply its place, Dr. I. M. Hayes is to make his monthly *News and Abstract* a weekly journal. He has visited the city here and secured some persons who will furnish him with the society news here. Some of those who were to have been connected with the proposed New York weekly will now work for the new Philadelphia journal. Philadelphia has already three excellent medical periodicals, all standing well. It is not easy to see that there is room for another.

The number of students in the colleges here this winter is perhaps somewhat smaller than it was a year ago, but the difference is not great. Bellevue, which lost a good many students by its attempts to have a three-years course, has gained somewhat in numbers the present year. The College of Physicians and Surgeons fully holds its own under its changes. Considerable talk was made about the retirement of Dr. Thomas from the chair of obstetrics. This amounts to nothing, since he has not lectured on obstetrics for several years—Dr. McLean, his associate, doing all this. Dr. Thomas gave a series of didactic and clinical lectures on gynæcology. These were very popular, but chiefly so with students. Dr. T. is a very attractive speaker, and puts things in a very clear and telling manner. It is some time before one appreciates that he is telling you very elementary things.

There are a number of what might be called private clinics given in the city during the present winter. They are generally free, and are intended for the instruction of graduates rather than students. They are well attended, and are very useful. The lecturer is paid of course in the additional reputation and consultation practice that he gets. Dr. L. D. Bulkley gives a popular course on skin diseases at the New York Hospital. Dr. N. M. Shaffer also gives a very well-attended orthopœdic clinic at the New York Orthopœdic Hospital. This gentleman has shown himself to be an original and industrious worker. In a monograph on the hysterical element in orthopœdic surgery, published a couple of years ago, he added some very useful observations to the meagre literature of joint-neuroses, and his present lecture has laid down one rule with great emphasis. It is that the muscular spasm by which diseased joints are made more or less rigid is pathognomonic of an osteitis. It may be tested in the hip, for example, by strongly flexing the leg and thigh, then holding the pelvis firmly with one hand, and with the other rotating the thigh outwards, or gently flexing and extending it. There will be a spring-like resistance to any such attempts at movement, which is very characteristic. If a further test is required the patient is to be laid flat on the stomach. The pelvis is held with one hand and the knee grasped by the other. By lifting up the knee the psoas is put on the stretch. If there is osteitis the consequent muscular spasm will show itself very plainly. All these movements must be produced with the greatest gentleness. In a neurominosis there may be some such spasm, but it is not persistent. The disappearance of the gluteal fold indicates little.

The physiological facts regarding muscular tonus may not be out of place in this connection.

It used to be thought that the muscles of the body were kept in a state of constant but slight contraction by a stream of nerve impulses sent out from automatic centers in the spinal cord ; that the voluntary muscles through this means possess a *tonus*, just as the smooth muscles of the arteries do. The great proof of this was thought to be the fact, that when a muscle is cut across, the two cut ends retract. We now know that this phenomena is due chiefly to the striped muscles being elastic and, normally, slightly on the stretch. This is shown, in one way, by the fact that the muscles of the cadaver retract somewhat when cut, as do also those whose nervous supply is lost.

But, though there is plainly no physiological muscular tonus due to automatic spinal centers, there does seem to be a kind of reflex tonus, very slight in amount, perhaps, but of great physiological significance. Hang up a brainless frog by the jaw, having cut the sciatic nerve on one, say the right side only. It will be seen that the right leg hangs more loosely, and that the tip of the right foot hangs lower than the left. Now this same thing takes place when only the posterior (sensory) roots are cut, and also when only the skin is removed from the leg. Brondgeest has shown that by simply cutting the posterior roots that supply the sciatic the leg is lengthened. It is evident that the slight contraction or tonus of the muscles in the sound leg is due to a constant reflex excitation started at the periphery. This has been called the reflex tonus, and it is a different thing from the old simple muscular tonus. Its existence has a practical interest, inasmuch as it is concerned so greatly with the phenomena of disease, both as regards the points already referred to, and others. Pflüger, for example, has claimed for this reflex an importance in the production of animal heat. A large amount of heat is manufactured by the activity of the muscles. When at rest, of course very little chemical action goes on in them, and if they were perfectly at rest all the time a most important source of heat would be lost. The reflex tonus excites a slight degree of muscular activity which is enough to increase the heat production. If the irritation of cold air be applied to the skin, the reflex tonus is excited, and by increased chemical changes in the muscles, more heat is developed. It is in this way, in part, that ordinary falls in temperature do not affect the bodily temperature. *Per contra*, when the air is warm the reflex tonus (which Pflüger calls the chemical reflex tonus) is lessened in amount, and the muscles, being less excited, produce less heat.

NEW YORK CITY, Dec. 15, 1881.

